# A Longitudinal Study of Steller Sea Lion Natality Rates in the Gulf of Alaska with Comparisons to Census Data

**DOI:** 10.1371/journal.pone.0111523

**Published:** 2014-11-10

**Authors:** John M. Maniscalco, Alan M. Springer, Pamela Parker, Milo D. Adkison

**Affiliations:** 1 Department of Science, Alaska SeaLife Center, Seward, Alaska, United States of America; 2 Institute of Marine Science, University of Alaska Fairbanks, Fairbanks, Alaska, United States of America; Sonoma State University, United States of America

## Abstract

Steller sea lion (*Eumetopias jubatus*) numbers in the Western Distinct Population Segment are beginning to recover following the dramatic decline that began in the 1970s and ended around the turn of the century. Low female reproductive rates (natality) may have contributed to the decline and remain an issue of concern for this population. During the 2000s we found high natality among Steller sea lions in the Gulf of Alaska indicating a healthy population. This study extends these previous estimates over an additional three years and tests for interannual variations and long-term trends. We further examine the proportions of pups to adult females observed on the rookery and nearby haulouts during the birthing season to assess whether census data can be used to estimate natality. Open robust design multistate models were built and tested using Program MARK to estimate survival, resighting, and state transition probabilities in addition to other parameters dependent on whether or not a female gave birth in the previous year. Natality was estimated at 70% with some evidence of interannual variation but a long-term increasing or decreasing trend was not supported by the data. Bootstrap and regression comparisons of census data with natality estimates revealed no correlation between the two methods suggesting that census data are not an appropriate proxy for natality in this species. Longitudinal studies of individual animals are an appropriate method for estimating vital rates in species with variable detection over time such as the Steller sea lion. This work indicates that natality remains high in this region and is consistent with a population in recovery.

## Introduction

Over the past 2 centuries, several fur seal and sea lion (Otariidae) populations have recovered to healthy numbers following catastrophic collapses [1]. Steller sea lions (*Eumetopias jubatus*) in Alaska, USA may be emulating this trend as numbers over a large portion of the western distinct population segment (WDPS) have begun to increase in recent years following a precipitous 30-year decline that started in the early 1970s [2], [3]. Indeed, Steller sea lion populations may have undergone dramatic fluctuations in abundance several times during the past 4 millennia [4]. The most recent decline is primarily attributed to reduced juvenile survival during the 1980s in addition to minor reductions in reproductive rates and adult survival [5]–[7]. Changes in those vital rates could have resulted from nutritional stress, killer whale (*Orcinus orca*) predation, or a combination of these and other factors such as direct interactions with fisheries [8]–[11]. The latest upswing in Steller sea lion numbers was estimated to have begun in the early 2000s [12] and may be attributed to improved juvenile survival [13] and high natality rates that are reflective of a healthy population [14]. However, some work based on population demography and theoretic modeling has suggested natality reached an all-time low during the past decade [15].

Continued monitoring of vital rates is essential for species of concern such as the WDPS of Steller sea lions, which is classified as endangered under the Endangered Species Act. Of the two approaches to obtaining vital rate information, longitudinal, individual-based studies have many advantages over cross-sectional, population-level studies [16]. Yet, published estimates of vital rates based on longitudinal studies are rare for the WDPS of Steller sea lions even though hundreds of millions of dollars have been dedicated to researching these animals. Here we provide an update of our previous work [14] on natality rates for these animals in the Gulf of Alaska based on a longitudinal study of adult females and assess the potential for using census data (counts) to predict trends in natality over time.

Natality is defined as the birthrate or the proportion of births to some segment of the general population – generally mature females. Female Steller sea lions become reproductively mature at three to seven years of age and give birth to one pup per year but not necessarily every year [17]. The birthing season typically ranges between late May and early July in Alaska [18]. Twinning is extremely rare [19] and juveniles are often weaned during the spring months at age 1, age 2 or even older [13], [20]. However, natality does not include neonatal or juvenile survival, and it is important to differentiate these life history variables because of the widely different factors that may affect them. Low rates of natality among pinnipeds have been related to disease, contaminants, and poor nutrition [6], [21]; whereas neonatal or juvenile survival may be more influenced by weather conditions, predation, or food limitation [22]–[24]. By lumping life history stages together, information pertinent to understanding population dynamics can be hidden or lost entirely.

We previously outlined several reasons why it may be difficult to estimate natality based on proportions of pups on rookeries and haulouts late in the breeding season [14]. Primarily, these are uncertainties about the extent of neonatal mortality due to storm waves for example, and proportions of females foraging at sea which may change systematically with shifting oceanic regimes [14]. Assuming stability in these variables over time might lead to erroneous conclusions about the proportion of pups that are born to a population of sea lions. We further suggested that disparities in natality estimation techniques may be resolved by long-term comparisons of census data with direct estimates of natality based on longitudinal observations of adult females and mark-recapture modeling [14]. In this study, we added 3 additional years to our previous time series and extended our natality estimates over a 10-yr period, 2003–2012, using robust-design mark-recapture statistics. We further compared these results to census counts of the proportion of pups to adult females on rookeries and haulouts to determine if census data can be used to predict trends in natality. If proportions of pups to adult females, or non-pups, can be used to predict natality, we should expect positive correlations between these proportions and mark-recapture estimates of natality over time.

## Materials and Methods

### Ethics Statement

This research was conducted in accordance with Alaska SeaLife Center Institutional Animal Care and Use Committee Protocol No. R10-03-01 and National Marine Fisheries Service Permit No. 14324 for research on endangered Steller sea lions. The Chiswell Island group is part of the U. S. Fish and Wildlife Service National Maritime National Wildlife Refuge. Research was conducted on refuge lands under Right-of-Way Permit No. M-344-AM and Special Use Permit No. 74500-10-001 and earlier versions.

### Study Site and Observational Methods

**Figure 1:**
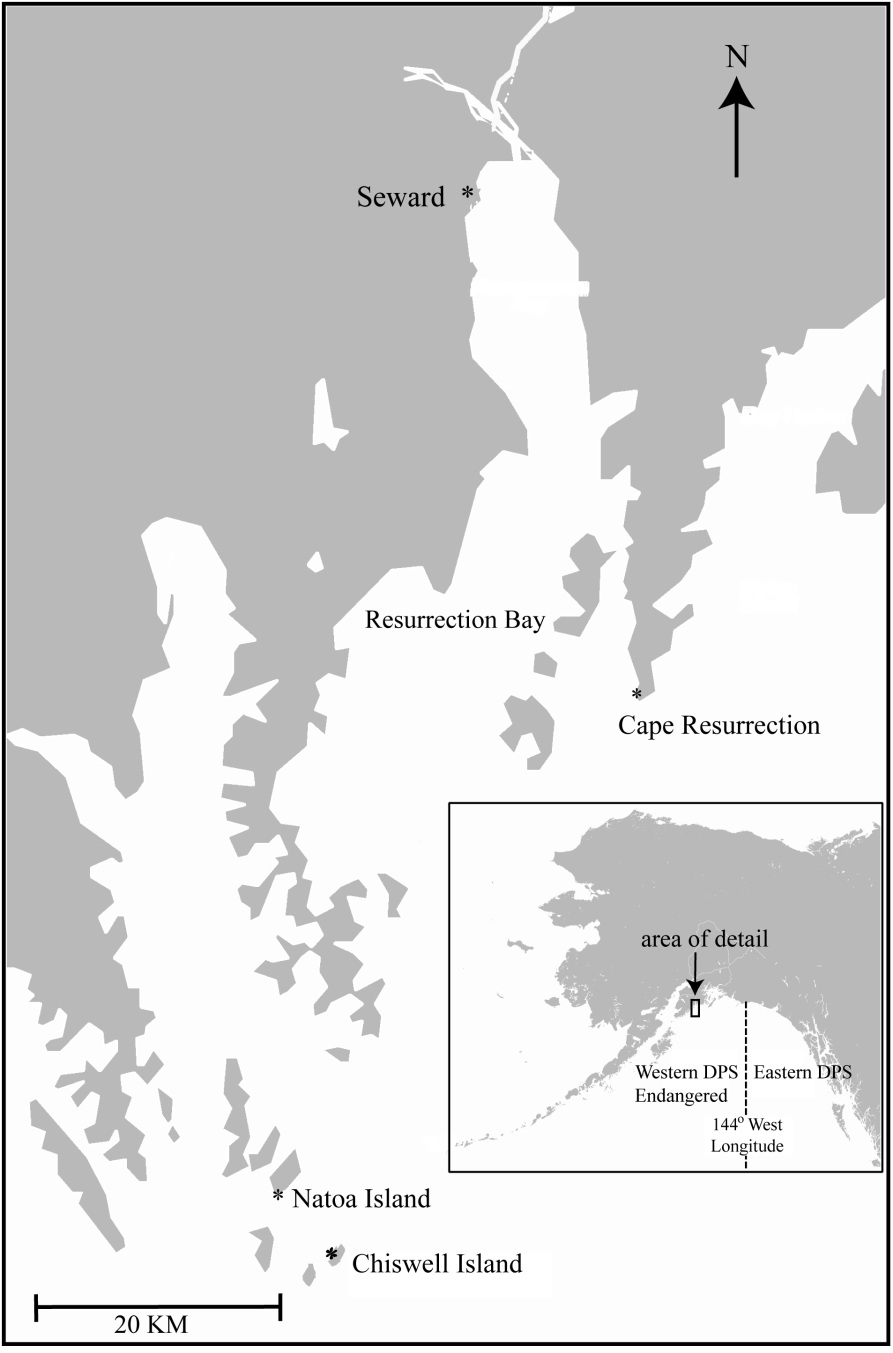
Location of the Chiswell Island Steller sea lion rookery and remotely monitored haulouts at Cape Resurrection and Natoa Island in Kenai Fjords, Gulf of Alaska.

The focal area of this research included the Steller sea lion rookery on Chiswell Island (59° 59.18′ N, 149° 23.40′ W) and nearby haulouts in Kenai Fjords (Figure 1) which lie within the range of the endangered WDPS. The population decline at the Chiswell rookery was similar to that of other rookeries in the central Gulf of Alaska – that is, abundance fell by 90% from 1,106 adults in 1976 [25] to approximately 90 adults and 50–80 pups in the 2000s [26]. Beginning in 1999, up to six remotely operated video cameras were used to monitor Steller sea lions (see [26] for details). Video images, which provided complete spatial coverage of the Chiswell Island rookery, were viewable and controllable in real-time from the Alaska SeaLife Center 65 km away. Cameras were also installed and monitored at nearby haulouts beginning in 2000 (Figure 1).

Most adult Steller sea lions can be individually identified by unique scars, fungal patches, and/or flipper patterns, and longitudinal studies have been successfully conducted on animals identified by such means [23], [24], [26], [27]. Over the course of this study, female sea lions with unique markings (n = 184) were tracked and digital photos of these animals and their distinguishing marks were taken on a regular basis (at least twice per month) at all remotely-monitored sites in Kenai Fjords. Some breeding females were identified by flipper tags (n = 5) or brands (n = 16), and age was known only for these animals. Females that did not have at least two distinguishing marks and could not be reliably resighted from one year to the next were not used in the analysis. Although pictures and data for some females were collected as early as 1999, the period 1999–2002 was not considered in the analysis of natality rates because there was a more focused effort on sighting females giving birth over females that did not. All females with unique markings were tracked from 2003 onward whether or not they gave birth.

Our observations were conducted year-round but intensified during the birthing and breeding season beginning in mid-May. Hour-long scan sampling for identifiable females and their pups was conducted four to ten times daily from 0600 h to 2200 h; earlier and later hours were added around the summer solstice (21 June) when light levels were sufficient for viewing sea lions. After 10 August, observations were recorded from approximately sunrise to sunset as diminishing daylight allowed. Events such as births and deaths were opportunistically recorded as they occurred or within 4 hrs of their known occurrence [24], [26]. Births that happened overnight were recorded the following morning as having occurred at the half-way point of non-observation hours.

Complete census counts of all sea lions by age class (adult males, adult females, juveniles 1–4 yrs old, and pups 0–1 yrs old) on the rookery and at two nearby haulouts (Figure 1) were made at approximately 1100 h throughout the breeding season. Ratios of pups to adult females on the rookery and haulouts were summarized between 25 June and 15 July each year to determine if these counts could be used as a proxy for natality rates. This date range corresponds to when the National Marine Fisheries Service conducts their range-wide flight surveys for population counts [2], [28] which are used in some models for vital rates estimation [7], [15]. In some cases when census counts were not conducted (≤2 each yr; 14 out of 208 total), daily numbers of each age class were interpolated from previous and subsequent counts.

Steller sea lion mothers in the WDPS will normally remain with their newborn pups for 8 to 12 days following parturition [26], [27]. Given the duration and detail of observations in this study (frequent scans and complete spatial coverage of the rookery), it is highly unlikely that any births went unnoticed. Furthermore, females that give birth to stillborns are not normally considered to be productive nor are stillbirths considered in the definition of natality. However, we include full-term stillbirths in our analysis (approximately 2%; [24]) so that our estimates are comparable to historic estimates during the 1970s when the population was presumed healthy, and in the 1980s during the height of the decline in this region. Those earlier estimates were based on late-term pregnancies of adult females collected in the field and could not account for stillbirths [6]. In addition, those previous studies only considered reproductively mature females whose status was known by examination of copora lutea in ovaries [6], [17]. It was neither possible nor the intention of this study to verify reproductive maturity for females that were not observed to give birth even when age was known. Therefore, females of known age were included in this study beginning at 5 years of age to be consistent with the average age of sexual maturity of 4.6 yrs [17], which would indicate that age of first pupping would be at about 5.6 yrs. Females of unknown age were included in this study if they were judged to be at least 4 yrs of age as approximated by visual size comparisons to other known-age females lying nearby.

Non-breeding haulouts within the study area were also monitored during the birthing season to account for females that may have spent more time at those locations and to reduce sample bias toward more fecund females that may spend more time at the rookery seasonally and across years. Females at haulouts were included in the analyses if they met the aforementioned sighting and maturity criteria. Adult females that were observed without a pup during any birthing season were classified as not giving birth whether on the rookery or a haulout. However, some of the animals in the Chiswell Island population that were not giving birth on the rookery in any given year spent the summer elsewhere, presumably outside of the study area. Adult females that returned to the study area later in the year were typically without a pup but a few (<2%) did return with a suckling pup indicating that they gave birth elsewhere. Therefore, females that were without a pup when observed only during the non-pupping season could not be defined with certainty as not giving birth during the previous summer. We were able to account for this uncertainty by modeling the data as a hidden Markov process using a multi-state robust design [29].

### Data Analysis

Open robust design (ORD) multi-state models with state uncertainty and seasonal effects were constructed and run using Program MARK [30]. Data were modeled across 10 primary periods (years 2003–2012) and 4 secondary periods – summer (Jun–Aug), autumn (Sep–Nov), winter (Dec–Feb), and spring (Mar–May) for a total of 40 sampling occasions. For each occasion, the states of every female were recorded as “b” – observed birth or with pup, “n” – observed but did not give birth, “0” – not observed, and “u” – observed during the non-birthing season without a pup, state uncertain. ORD models allow for entry and departure from the study area between primary periods and secondary periods, as often occurs among sea lions between seasons, but these models also greatly increase the number of parameters that can be estimated compared to models without a robust design. Nevertheless, robust design models allow estimation of temporary emigration from the study area and provide more precise estimates of survival and state transitions [29], [31], [32]. Furthermore, multistate ORD models do not necessarily require that the state of females be known with certainty or that females with and without a pup have equal sighting probabilities [31], [33]. A goodness of fit test for multi-state ORD models when both states are observable has not been developed. However, a goodness of fit test using a simpler modeling approach for much of these data has shown only a slight and insignificant degree of overdispersion [14] and we have no reason to believe that data in the current model structure are much different.

The following parameters were estimated for this study with some initial constraints that were appropriate for the behavioral biology of Steller sea lions and to keep model run times reasonable:

- S*_i_* ^x^ = probability that a female in state x in year *i* survives until *i*+1.
- p*_i_* ^x^ = probability that a female is sighted in time period *i* in state x, given that she is present in the study area in period *i*. For secondary sampling periods a scaled covariate was added to reflect seasonal changes in observer effort which was highest during summer and lowest during winter.
- ψ*_i_* ^xy^ = transition probability or probability that a female in state x in year *i* is in state y in year *i*+1, given that she survived from year *i* to *i*+1.
- π_t_^x^ = probability that a female is first observed in state x in year *i* for years 2–9 of the study.
- ω_t_^x^ = probability that a female is in state x in year *i*, used as an estimate of natality in this study.
- δ_t_^x^ = probability that a female is classified correctly to state. Constrained to be equal across all times periods for females with a pup. Constrained to be equal for females not seen with a pup across primary periods but allowed to vary between 1^st^ secondary period (summer) and 2^nd^–4^th^ secondary period combined.
- pent_t_^x^ = probability that a female in state x enters into the study area between a given secondary period. Constrained to be equal across primary periods for both states but allowed to vary between states and secondary periods with the mlogit link function to sum to ≤1.
- d_t_^x^ = probability that a female in state x departs from the study area between secondary periods. Constrained to be equal across primary periods but allowed to vary between states and between secondary periods.
- α_t_^x^ = probability that the attribute for state assignment has appeared (i.e., pup). Constrained to 1 for the 1^st^ secondary period and to 0 for all other sampling periods because females only give birth during summer.
- c_t_^x^ = probability that the attribute ceases – i.e., pups are weaned. Weaning often occurs during the spring months (Trites et al. 2006) and therefore was fixed to 0 for the 1^st^ and 2^nd^ secondary period intervals and allowed to vary during the 3^rd^ interval.

These model parameters were combined across primary and secondary sampling periods into a joint multinomial likelihood using the mlogit link function in Program MARK. Additional constraints were placed on the models with regard to biological relevance in the search for parsimony. For example, survival (S) and state transitions (ψ^xy^) were constrained to be equal over time in some models to assess whether the data more closely fit those model structures compared to time-varying model structures. The average ratio of pups to adult females on Chiswell Island and nearby haulouts was included as an annual covariate in some models to assess the effect on model fit. In the same manner, the total numbers of pups born on Chiswell each year was included as annual covariate for female state (ω) in addition to modeling linear trends. All models were compared with Akaike’s Information Criteria (AIC; [34]), corrected for small sample bias (AICc; [35]). AICc weights, calculated from model differences in AICc values (ΔAICc), indicated relative support for the various models. Likelihood ratio tests (LRT) were also run to test for meaningful differences in models of interest. Finally, all models were averaged (multimodal inference; [36]) for estimates of parameter values relevant to this study.

We further assessed the possibility of using ratios of pups to females observed on rookeries and haulouts as a proxy for natality rates. Census data were log transformed to approximate normality and the ratios, *ln* (pups)/*ln* (adult females), were regressed against natality rates estimated from the ORD analysis for each year. This was conducted for each of 21 days between 25 June and 15 July each year to determine if any of these days provide a more accurate representation of natality over other days in that time period. We also conducted a bootstrap randomization test on standardized census data. The pup/adult female ratio was standardized to remove intra-seasonal patterns of haulout use by subtracting the daily means across years and adding back the overall mean to the daily count. We ran 1000 iterations to compare with estimated natality rate for the corresponding year. If count ratios are representative of natality rates across years, we would expect to find a significant correlation between these variables.

## Results

A total of 16 models were run and ranked in order of lowest AICc score (Table 1). Models with no time differentiation in survival (S), sighting probability (p), and state transitions (ψ) showed more support for the data compared to those with variation across years (primary sampling periods). Time variation across seasons (secondary periods) was retained for sighting and departure (d) probabilities due to seasonal movements into and out of the study area (Alaska SeaLife Center, unpublished data), and the desire to model these changes. There was some evidence of differences in survival between females giving birth (89.0±1.4%) and those that did not (86.1±2.4%) as variation in this state was expressed in two of the four best models with no significant differences (Table 2; LRT: Model 1 vs 2: χ^2^ = 1.602; *P* = 0.206; Model 3 vs 4: χ^2^ = 1.107; *P* = 0.293). There was also little evidence of annual variation in the proportion of females giving birth (ω^b^) as noted in a comparison of Models 1 and 3 (Table 1; LRT: χ^2^ = 16.940; *P* = 0.050) and Models 2 and 4 (Table 1; LRT: χ^2^ = 16.444; *P* = 0.058). See also Table 2 for time varying differences in ω^b^ for models 3 and 4.

**Table 1:** Comparison of models tested using a multi-state open robust design in Program Mark for estimation of survival (S), sighting probabilities (p), state transitions (ψ), and state occupation (ω) among Steller sea lions in Kenai Fjords.

Model Rank	Model Structure	No. Param.	AICc	ΔAICc	AICc Weights	Model Likelihood
**1**	S_._ p_st.2°_ ψ_st._ π_._ ω_._ d_st.2°_	30	6106.846	0.000	0.4067	1.0000
**2**	S_st._ p_st.2°_ ψ_st._ π_._ ω_._ d_st.2°_	31	6107.313	0.467	0.3220	0.7916
**3**	S_._ p_st.2°_ ψ_st._ π_._ ω_t_ d_st.2°_	39	6108.617	1.771	0.1678	0.4125
**4**	S_st._ p_st.2°_ ψ_st._ π_._ ω_t_ d_st.2°_	40	6109.601	2.755	0.1026	0.2522
**5**	S_._ p_st.2°_ ψ_st._ π_._ ω_p_ d_st.2°_	39	6119.334	12.488	0.0008	0.0019
**6**	S_st._ p_st.2°_ ψ_st._ π_._ ω_r_ d_.2°_	39	6124.224	17.378	0.0001	0.0002
**7**	S_st._ p_st.2°_ ψ_st._ π_t_ ω_t_ d_st.2°_	47	6124.259	17.413	0.0001	0.0002
**8**	S_st._ p_st.2°_ ψ_st._ π_._ ω_r_ d_st.2°_	40	6125.533	18.687	0.0000	0.0001
**9**	S_.t_ p_st.2°_ ψ_st._ π_t_ ω_t_ d_st.2°_	54	6129.711	22.865	0.0000	0.0000
**10**	S_st._ p_st.2°_ ψ_st._ π_._ ω_up_ d_st.2°_	40	6133.919	27.073	0.0000	0.0000
**11**	S_st._ p_st.2°_ ψ_st._ π_._ ω_dn_ d_st.2°_	40	6136.540	29.694	0.0000	0.0000
**12**	S_st._ p_st.2°_ ψ_st._ π_._ ω_p_ d_st.2°_	40	6136.540	29.694	0.0000	0.0000
**13**	S_st._ p_st.2°_ ψ_st._ π_._ ω_t_ d_.2°_	37	6138.479	31.634	0.0000	0.0000
**14**	S_st.t_ p_st.2°_ ψ_st._ π_t_ ω_t_ d_st.2°_	63	6139.086	32.241	0.0000	0.0000
**15**	S_st.t_ p_st.2°_ ψ_st.t_ π_t_ ω_t_ d_st.2°_	79	6156.477	49.632	0.0000	0.0000
**16**	S_st.t_ p_st.1°.2°_ ψ_st.t_ π_t_ ω_t_ d_st.2°_	151	6190.485	83.639	0.0000	0.0000

Proportion of the population released in each state (π) and departure probabilities (d) were also manipulated to test their effects on model fit. Initial constraints on parameters not listed here are outlined in the Methods.

*Notes*: Subscripts indicate state variation (st; with vs without pup), time variation (t), and increasing (up) or decreasing (dn) linear trends across primary periods in addition to time variation specific to primary periods (1°) and secondary periods (2°). Additional subscripts identify annual covariates of total number of pups born (p) and the average ratio of pups to adult females (r) observed.

**Table 2:** Estimates (± SE) from the four top-ranked models in Table 1 for survival of females that gave birth (S^b^) and those that did not (S^n^), occupation of birthing state (ω^b^) in any given year, and sighting probabilities by season for females that gave birth (p^b^) and those that did not (p^n^).

Parameter	Model 1	Model 2	Model 3	Model 4
S^b^	88.1% (1.2%)	89.0% (1.4%)	88.1% (1.2%)	89.0% (1.4%)
S^n^	88.1% (1.2%)	86.1% (2.4%)	88.1% (1.2%)	86.1% (2.4%)
ω^b^–2003	70.5% (1.6%)	70.5% (1.6%)	85.5% (5.0%)	85.5% (5.0%)
ω^b^–2004	70.5% (1.6%)	70.5% (1.6%)	75.3% (5.7%)	75.3% (5.7%)
ω^b^–2005	70.5% (1.6%)	70.5% (1.6%)	60.0% (5.7%)	60.0% (5.7%)
ω^b^–2006	70.5% (1.6%)	70.5% (1.6%)	67.6% (5.4%)	67.6% (5.4%)
ω^b^–2007	70.5% (1.6%)	70.5% (1.6%)	75.5% (4.7%)	75.5% (4.7%)
ω^b^–2008	70.5% (1.6%)	70.5% (1.6%)	68.2% (4.8%)	68.2% (4.8%)
ω^b^–2009	70.5% (1.6%)	70.5% (1.6%)	71.8% (4.6%)	71.8% (4.6%)
ω^b^–2010	70.5% (1.6%)	70.5% (1.6%)	64.7% (4.9%)	64.7% (4.9%)
ω^b^–2011	70.5% (1.6%)	70.5% (1.6%)	76.6% (4.2%)	76.6% (4.2%)
ω^b^–2012	70.5% (1.6%)	70.5% (1.6%)	66.3% (4.6%)	66.3% (4.6%)
p^b^–summer	99.9% (<0.1%)	99.9% (<0.1%)	99.9% (<0.1%)	99.9% (<0.1%)
p^n^–summer	98.3% (0.9%)	97.7% (1.2%)	97.7% (1.2%)	97.7% (1.3%)
p^b^–fall	94.6% (1.8%)	94.6% (1.8%)	94.6% (1.8%)	94.6% (1.8%)
p^n^–fall	74.3% (4.9%)	72.8% (4.4%)	72.9% (4.4%)	72.8% (4.4%)
p^b^–winter	24.6% (3.6%)	24.6% (3.6%)	24.6% (3.6%)	24.6% (3.6%)
p^n^–winter	12.7% (2.3%)	14.3% (4.0%)	14.3% (4.0%)	14.3% (4.0%)
p^b^–spring	70.4% (4.1%)	70.4% (4.1%)	70.4% (4.1%)	70.4% (4.1%)
p^n^–spring	39.4% (4.2%)	40.1% (4.7%)	40.1% (4.7%)	40.1% (4.7%)

An *a priori* decision was made to test for an increasing trend in natality, as the population in this region had been increasing over the study period [2]. However, fitting a linear increasing trend to natality did not improve model fit (Model 10 vs Model 4). Upon examination of natality estimates from the top ranked models, it was determined that a linear decrease in natality should also be tested for, but this also did not improve model fit (Model 11). We further tested for an improvement in model fit using either the total number of pups born or the average ratio of pups to adult females as linear covariates for ω^b^ each year. Both greatly reduced model fit compared to the model with full time dependence in ω^b^ (Models 5 & 6 vs Model 3 and Models 8 & 12 vs Model 4).

**Figure 2:**
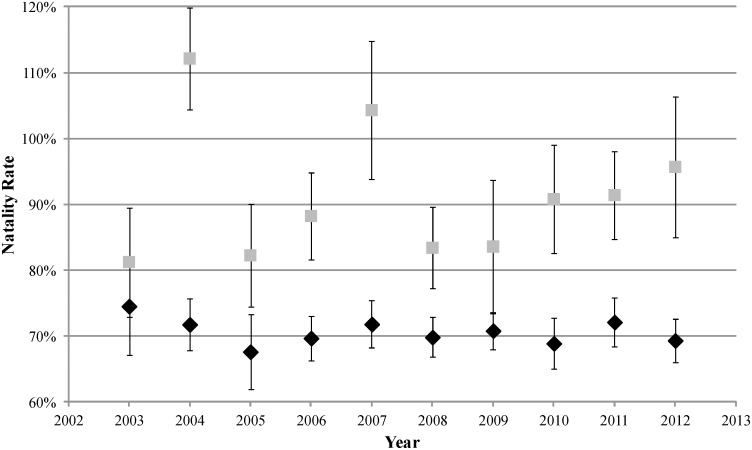
Mark-recapture estimates of natality rates (black diamonds) and natality estimates based on census data (gray squares) for adult female Steller sea lions at the Chiswell Island rookery from 2003 to 2012 with 95% confidence intervals. Note that the mark-recapture estimates are different from estimates expressed in Table 2 due to model averaging.

The four best models held 99.9% of the total weight (Table 1) and therefore, model averaged parameter estimates were based primarily on these structures. Survival to a subsequent year for females giving birth (S^b^; 88.49±1.33% SE) was slightly higher than for those that did not give birth (S^n^; 87.27±2.06%). Sighting probabilities were higher for females giving birth (p^b^) compared to those without a pup (p^n^) and seasonal sighting probabilities were especially low during winter (Table 2). Natality rates (ω^b^) were high with an overall average of 70.5±1.6% that ranged narrowly from 67.6%±5.7% to 74.5%±7.3% (Figure 2). Also, females that gave birth in year *i* were nearly as likely to give birth in year *i*+1 (ψ^bb^ = 0.708±0.021) as females that did not give birth in year *i* (ψ^nb^ = 0.736±0.033).

**Figure 3:**
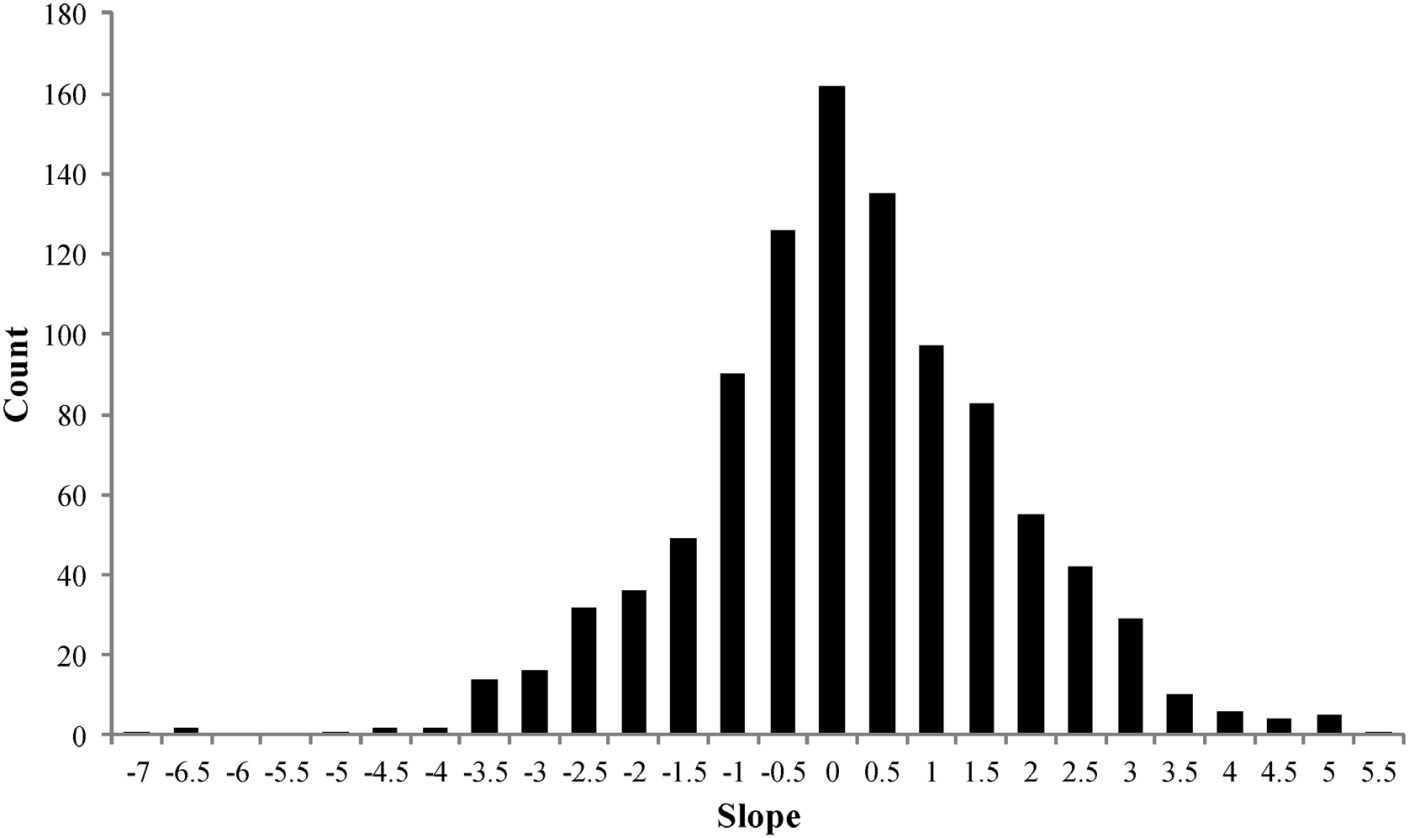
Bootstrap histogram of regression slopes based on pup to adult female ratios versus mark-recapture natality estimates. This figure shows that the regression slopes were strongly centered around zero with no correlative trend.

Natality based on the ratio of pups to adult females on the rookery and nearby haulouts was higher than estimates from the mark-recapture modeling and had greater variation (91.3%±3.2%; Figure 2). Estimates for some years were unrealistically >100% because varying numbers of adult females were foraging at sea and unavailable to be counted. However, the intent of this analysis was not to obtain exact estimates of natality but to determine if natality rates based on census counts are correlated with direct estimates of natality and could therefore be used as a meaningful proxy for changes in natality. To test for a correlation, those rates were regressed against the natality rates (ω^b^) estimated from Program MARK for each year. Slopes of the 21 regressions ranged from −0.250 to +0.223 and none were significant (*P*-value range: 0.080–0.925). The bootstrap analysis of the pup/adult female ratios also resulted in a non-significant relationship with natality (r^2^ = 0.048; *P* = 0.542) and had slopes centered around zero (Figure 3).

## Discussion

### Mark-Recapture Parameter Estimates

We previously examined natality of Steller sea lions in this region over seven years using a standard mark recapture multistate approach without robust design [14]. The current analysis was undertaken to improve our estimates using the robust design, to examine possible interannual and long-term changes in natality, and to assess the use of census data as a proxy for natality.

Not surprisingly, estimates of natality were similar to those previously found in this region at about 70% but with a smaller standard error (1.6 vs. 2.5%; [14]), which may be due partly to the larger sample size in this study. In addition, parameter estimates such as these are notably improved using the robust design as reported by others [29], [32]. Sighting probabilities were high during the summer months for females giving birth and for those not giving birth in any given year (Table 2). Yet many non-breeders were not observed on the rookery during the birthing season (mid-May to Mid-July) and arrived later in the summer or autumn. If we only considered sightings during the birthing season much of the information about non-breeders would be lost and hence lead to greater uncertainty in our estimates.

Natality was not much different from pre-decline levels (67%) but better than estimated during the height of the population decline (55%) in the Gulf of Alaska during the 1980s [6]. It has previously been suggested that natality rates bottomed out at about 43% in the early 2000s, as estimated from an inferential population dynamic model [15]. A rapid change in natality from percentages in the mid-40s to nearly 70% would seem quite unusual, hence it was of interest to test for an increasing trend in this study. However, the model we tested with a increasing trend in natality did not fit the data, nor did one with a decreasing trend. In our analysis, natality ranged narrowly around 70%, with 2 of the 4 best models supporting interannual fluctuations but no trends. Therefore, we have little evidence to support unusually low natality rates during any year of this study.

Notwithstanding variation in survival, natality rates of 60% to 75% have been generally associated with stable or increasing populations of pinnipeds [37]–[40], including the Eastern Distinct Population Segment (EDPS) of Steller sea lions [41]. Natality of 55% or lower has been associated with declining populations of otariids and related to the adverse effects of density dependant factors such as intraspecific competition for food or breeding space [6], [42] but see also [43]. Therefore, our estimate of natality from the Chiswell Island population of Steller sea lions is indicative of a population where adult females seemingly are not under resource limitation. Populations in this region and as far west as the eastern Aleutian Islands have been increasing since the early 2000s [2]. We cannot say with certainty that natality rates estimated here in the eastern Gulf of Alaska are representative of natality as far west as the eastern Aleutian Islands, but given the similar population trends [2], we currently have no reason to suppose otherwise.

During periods of food limitation long-lived mammals may exhibit a cost of reproduction in terms of reduced probability of survival or reproduction in successive years compared with times of food abundance [44], [45]. Such a cost likely affected Steller sea lions during the 1980s when pregnancy was negatively correlated with lactation status [6]. Similar to our previous work [14], we found no evidence of a cost of reproduction among Steller sea lions during the period of this study (2003–2012) with survival and subsequent reproduction not being correlated with previous birthing status. This finding is consistent with a healthy population that is not exhibiting signs of broad scale food limitation [8], [45], [46].

### Comparisons with Census Data

Long-term census-based studies provide important information regarding changes in demographics, distribution, densities, and trends of wildlife populations including the potential influences of climate change and human activities [16]. However, using census data to estimate vital rates in variably detectable species such as pinnipeds that spend a large proportion of their time foraging at sea may be difficult. Using several methods, we were unable to find a correlation between either pup counts or pup to female ratios and our natality estimates. As our natality estimates showed little year to year variation, this could have made such relationships difficult to detect. Nevertheless, if census counts can be used as a proxy for natality, we could expect at least weak positive correlations, but they were strongly centered around zero (Figure 3).

Using census counts for modeling vital rates is unusual for many large vertebrates and caution is warranted when modeling such data for vital rate estimates [47]. Census counts of Steller sea lions can vary with tide height, storm waves, time of day, and food availability [48]–[50]. Pup counts may or may not reflect the actual number of births because storm waves during the peak of birthing are variable across years and can cause huge losses of neonates [23], [24]. These variations are typically assumed to be consistent over time [7], [15] but they are not [14]. This is a primary reason why census data cannot be reliably used to estimate vital rates in species such as Steller sea lions unless adjustments are made to the data that appropriately reflect environmental stochasticity over time.

Accounting for variation in sighting probabilities is integral to longitudinal studies employing mark-recapture analysis. Long-term studies of a representative sample of individuals are necessary for accurate estimates of age-specific survival and natality, but are invariably more time consuming and costly than snapshot population counts [16], [51]. Nevertheless, these mark-recapture methods provide critical information for understanding life histories and behavior of species of concern. In addition to natality, survival, and sighting probabilities, many other parameters can be estimated with the mark-recapture data analysis techniques currently available [29], [32], [33]. For instance, longitudinal studies of branded Steller sea lions are providing insights into temporary and permanent migration between the distinct population segments [52]. Without such work, we might mistakenly assume that populations receiving such immigrants were increasing solely due to improved survival or natality.

Census counts provide information on population trends over time, whereas individual-based longitudinal studies will more specifically inform researchers of why and how populations are changing. Based on census count data, we know that the EDPS of Steller sea lions has been increasing over the past 30 years [53]. Based on longitudinal studies we also recognize that the population increases likely result from a combination of good juvenile survival [54], good natality [41], and some immigration from the WDPS [51]. All of this information and more has lead to the recent delisting of the EDPS from the Endangered Species List [55]. The WDPS remains endangered, but with population trends continuing to increase, downlisting to threatened status is possible by 2015 [2]. Current population growth in the WDPS can be attributed to good natality and adult survival ([14]; this study) in addition to improved juvenile survival [13] since the height of the decline. Continued monitoring of vital rates is essential to detect changes that could threaten recovery of the species. Longitudinal studies of this nature can detect changes in vital rates that may happen quickly and drastically in response to increasing environmental stochasticity in the face of a shifting global climate [56].
